# Method Development for Multimodal Data Corpus Analysis of Expressive Instrumental Music Performance

**DOI:** 10.3389/fpsyg.2020.576751

**Published:** 2020-12-04

**Authors:** Federico Ghelli Visi, Stefan Östersjö, Robert Ek, Ulrik Röijezon

**Affiliations:** ^1^ Gesture Embodiment and Machines in Music (GEMM), School of Music in Piteå, Luleå University of Technology, Luleå, Sweden; ^2^ Division of Health, Medicine and Rehabilitation, Department of Health Sciences, Luleå University of Technology, Luleå, Sweden

**Keywords:** embodied music cognition, movement analysis, chunking, stimulated recall, coarticulation, expressive music performance, multimodal analysis

## Abstract

Musical performance is a multimodal experience, for performers and listeners alike. This paper reports on a pilot study which constitutes the first step toward a comprehensive approach to the experience of music as performed. We aim at bridging the gap between qualitative and quantitative approaches, by combining methods for data collection. The purpose is to build a data corpus containing multimodal measures linked to high-level subjective observations. This will allow for a systematic inclusion of the knowledge of music professionals in an analytic framework, which synthesizes methods across established research disciplines. We outline the methods we are currently developing for the creation of a multimodal data corpus dedicated to the analysis and exploration of instrumental music performance from the perspective of embodied music cognition. This will enable the study of the multiple facets of instrumental music performance in great detail, as well as lead to the development of music creation techniques that take advantage of the cross-modal relationships and higher-level qualities emerging from the analysis of this multi-layered, multimodal corpus. The results of the pilot project suggest that qualitative analysis through stimulated recall is an efficient method for generating higher-level understandings of musical performance. Furthermore, the results indicate several directions for further development, regarding observational movement analysis, and computational analysis of coarticulation, chunking, and movement qualities in musical performance. We argue that the development of methods for combining qualitative and quantitative data are required to fully understand expressive musical performance, especially in a broader scenario in which arts, humanities, and science are increasingly entangled. The future work in the project will therefore entail an increasingly multimodal analysis, aiming to become as holistic as is music in performance.

## Introduction

This paper discusses method development for multimodal research on expressive music performance. We report on a pilot study, carried out by Gesture Embodiment and Machines in Music (GEMM), a cross-disciplinary research cluster, together with members of the Norrbotten NEO^1^ – a professional contemporary music ensemble, part of the research environment at the Luleå University of Technology. The study constitutes the first step in the development of a comprehensive approach to the understanding of music performance as a multimodal experience. We aim at bridging the gap between qualitative and quantitative approaches by combining methods for data collection, with the purpose of building a data corpus containing multimodal measures linked to high-level subjective observations. This will allow for a systematic inclusion of the knowledge of music professionals in an analytic framework, which synthesizes methods across established research disciplines. As proposed by Lesaffre and Leman (2020, p. 3) such interdisciplinary entanglements between arts, humanities, and science demand a coupling requiring “open flows of information, which copes with important transformations regarding how science works, as well as how companies and societies innovate.” Along these lines, the presence of Norrbotten NEO in the heart of the research cluster represents a novel potential but also poses central questions regarding the development of methods for multimodal research on expressive music performance. The shift toward a true entanglement of arts and science demands new forms for qualitative data collection. In this paper, we report on the initial explorations of how professional musicians can obtain an integrated role in the generation of several layers of qualitative data, and we consider how such materials can be further analyzed through the use of quantitative methods.

In the remaining subsections of the introduction, we provide a theoretical background to the research. In section Qualitative Analysis, we outline the forms of qualitative analysis applied in the study. In section Quantitative Analysis, we provide a brief backdrop of the quantitative analysis of body movement in musical performance research. In section Knowledge Gaps, we identify the knowledge gaps that the pilot study seeks to address. The design of the pilot study is described in section Design of the Pilot Study. Section Results of the Pilot Study presents the results of the pilot study starting with the quantitative data in section Identification and Extraction of Relevant Features. While the quantitative findings are limited, in section First-Person Observations and Cross-Comparison of Data we give a more substantial account of qualitative findings in the study and suggest some multimodal findings enabled by combining different modalities in the data. Finally, section Discussion and Future Work holds a discussion of these preliminary findings in the pilot study and how these may be taken further in future work.

### Music Performance and Embodied Cognition

The notion of embodiment entails a phenomenological and biological grounding of human cognition and experience of the world in action (Clayton and Leante, 2013). This perspective has notably shifted scholarly understandings of musical perception.

According to the theory of embodied cognition, the sensorimotor system is central to all human thought-processes, which are “a product of the activity and situations in which they are produced” (Brown et al., 1989, p. 33). Thelen et al. (2001, p. 1) define embodied cognition as dependent on “the kinds of experiences that come from having a body with particular perceptual and motor capacities that are inseparably linked and that together form the matrix within which memory, emotion, language and all other aspects of life are meshed.” A fundamental aspect of these “perceptual and motor capacities” is discussed in neuroscience as the coupling of action and perception. Leman describes this coupling as the interaction between mechanisms taking place in different layers of the body (Leman, 2012). The body image may be thought of as the explicit understanding that we have of our own bodies. It is an intentional state made up of several modalities: perceptual experiences of one’s own body; conceptual understandings of the body in general; emotional attitudes toward one’s own body (De Preester, 2007). At the level of body image, performative knowledge may be accessible through introspection and reflexive research methods, such as is common in autobiographical forms of artistic research. The body schema, on the other hand, involves “a system of motor capacities, abilities, and habits” (Gallagher and Cole, 1995) which operate largely subconsciously and constitute the greater part of what we may conceive of as a performer’s habitus. Gibson’s concept of affordances assumes a similar link between action and perception (Gibson, 1986). Taking the example of a musician, an instrument affords different musical possibilities to different performers; hence, the affordances of an instrument are as dependent on the individual performer as on the properties of the instrument.

### Motor Control in Music Performance

Learning and performing skilled movement tasks, such as playing a musical instrument, involves highly advanced sensorimotor control (Altenmüller, 2008). This includes sensory processing through proprioception, and the tactile, vestibular, visual, as well as, of course, the auditory systems. Human perception, through these sensory processes and the central nervous system (CNS), embraces both conscious and unconscious awareness of body position and movements, as well as of the task performance and the environment. *Via* feedback (reactive) and feedforward (anticipatory) control mechanisms, the CNS creates coordinated motor commands for well-adapted muscle activation (Franklin and Wolpert, 2011). Due to the time delay of sensory feedback, the CNS also uses an efference copy of the motor command in skilled fast movement performances. This efference copy is used to predict the results of the movement, already before sensory feedback has reached the CNS, and thereby allow for rapid actions and reactions needed in skilled motor tasks. The efference copy is also integrated with the sensory feedback, as a Kalman filter, to increase the accuracy of the estimation of the state of the body (Franklin and Wolpert, 2011). In well-coordinated movements, muscles, or part of muscles are either activated or inhibited in patterns of co-variation *via* neural motor commands from CNS, in order to skillfully achieve the desired goal of the task (Latash et al., 2007). Similarly, musical performance inherently involves well-adapted somatosensory synchronization (Repp and Su, 2013).

Skillful movements can be defined as the ability to accurately achieve the goal of a given motor task (i.e., accuracy), consistently during a high ratio of trials (i.e., with consistency or precision), and with an economy of effort (i.e., efficiency). This can, moreover, be achieved in various current and future contexts and environments (i.e., flexibility) and in relation to the individual’s capabilities and resources to effectively solve the motor task (Higgins, 1991). Skillful movements are achieved by adaptation and learning. Several classifications of the different learning phases have been proposed. A common classification includes three stages: (1) cognitive, (2) fixation, and (3) autonomous stages (Schmidt et al., 2018). In the first cognitive stage, the person has to solve what actions to take to achieve the goal. Various strategies are tried, where effective strategies are retained and ineffective strategies are discarded, and the performance is usually very inconsistent. The second fixation stage begins when the person has determined the most effective way of doing the task and starts to make smaller adjustments in how it is performed. Movement performance becomes more consistent. The third autonomous stage enters after a long time of practice. The skill can now be performed automatically without interference from other activities and simultaneous tasks, e.g., sight-reading while playing the clarinet a prima vista.

### Coarticulation, Chunking, and Segmentation in Music Performance

Theories of coarticulation, as a fundamental feature of human perception and production of speech, builds on the further observation of how language is made up of smaller components such as from word, to morpheme, to phoneme (Kühnert and Nolan, 1999). Hence, coarticulation conceptualizes how such components are woven together in the performance of language. The origin of coarticulation in the language is grounded in our embodiment: “The vocal tract is governed by the laws of physics and the constraints of physiology, but (also unlike the typewriter) it is producing its communicative artefact in ‘real time.’ It cannot move instantaneously from one target configuration to the next” (Kühnert and Nolan, 1999, p. 8, 9). Coarticulation is the result of the particular affordances of the vocal apparatus, which entails making a graceful movement from one phoneme to the next while projecting to the listener a coherent whole.

Similar processes of perceptual meaning formation have been observed in musical performance (sound-producing action) and perception (Godøy, 2014). Human perception of music builds on our ability for “chunking” audio signal in smaller units, on the level of phrase and sub-phrase (Godøy et al., 2010), but also, to weave these together into larger chunks through contextual smearing (Godøy, 2014). Coarticulation can be observed on different time scales. Many studies of coarticulation in music performance have focussed on what may be described as the prefix and suffix to a sound-producing action (see further Godøy, 2008), and hence, looking more at the anticipation of finger movement, for instance in piano playing (Engel et al., 1997). But coarticulation also plays an important role in the shaping of longer phrases and is reflected also in the temporal and spatial coarticulation of actions in multiple body parts. The identification of musical “goal-points” is, according to Godøy (2014, p. 540), based on “combined biomechanical, motor control, and perceptual constraints” and gives us intrinsic and “natural” criteria for chunking continuous streams of sound and gestures into meaningful units. Further, for Godøy (2006, p. 149), the theory of embodied music cognition suggests that these perceptual objects are not stored as “sound objects”; rather, he argues that “we actually recode musical sound into multimodal gestural-sonorous images based on biomechanical constraints (what we imagine our bodies can do), hence into images that also have visual (kinematic) and motor (effort, proprioceptive, etc.) components.” For instance, Godøy turns to Schaeffer’s observation of basic envelopes (dynamic shapes) of sound objects – impulsive, sustained, and iterative – and notes that these sound objects also have corresponding gestural types in the action of the performer. We found these observations of basic types of gestural sonic objects to be an important reference in the development of a multimodal framework for the analysis of music performance (see further below regarding the application of Laban Movement analysis (LMA) in the analysis of movement qualities in musical performance).

### Multimodal Music Representation and Analysis

Since multimodality has been identified as a central quality of musical experience, it is worth unpacking the term further. The word “multimodal” is used in various contexts. In psychology, neuroscience, and related disciplines, “modality” refers to a human sensory channel, and therefore the perception of stimuli that involve multisensory integration is referred to as “multimodal” (Small and Prescott, 2005). In music information retrieval (MIR) a “modality” is a source of musical information, such as audio, score, lyrics, video of a performance, etc. Thus, approaches that use multiple sources to represent and retrieve musical content are referred to as “multimodal” (Schedl et al., 2014). In human-computer interaction (HCI), multimodality occurs when the interaction between a user and a computer uses multiple means of input and output, e.g., speech recognition, touch, motion sensing, auditory feedback, etc. (Weiss et al., 2017). The definition of “multimodal” thus varies to some extent depending on the context in which the word is used. Yet, it essentially points to the experience or representation of something by means of multiple sources of heterogeneous nature.

A multimodal representation of a piece of music can contain several synchronized layers such as audio, symbolic representations (score, MIDI data), and audio descriptors (Briot et al., 2020); videos of the performance, physiological and motion data describing the performers’ movements; and semantic labeling and annotations describing expressivity and other high-level qualities of the music (Coorevits et al., 2016). The data contained in these concurrent layers can be used to individuate segments in the music, that is, parts that form its structural and temporal unfolding across multiple modalities. Different approaches to segmentation can help singling out and analyzing various musical elements: from single notes and acoustic components to phrases, gestures, chunks, and multimodal units of musical meaning such as gestural sonic objects (Godøy, 2018). Criteria for segmentation using quantitative data include onset detection in audio signals (Bello et al., 2005) or in physiological signals describing muscle activation (Solnik et al., 2008), and analysis of motion data for repetitive pattern detection and semantic clustering (Krüger et al., 2017). Qualitative approaches to segmentation include performer’s analysis of the score for the identifications of chunks (Östersjö, 2016) as well as observational analysis of video data through the use of open coding and stimulated recall (Coorevits et al., 2016). Through multimodal integration techniques – also known as multimodal fusion – processed audio, video, motion, and physiological signals can be further combined with symbolic and qualitative data in order to detect events useful for the analysis of musical content (Essid and Richard, 2012). These techniques are central for the development of machine learning models able to process and relate data from multiple modalities, and thereby gain an in-depth understanding of complex phenomena that humans experience multimodally (Baltrusaitis et al., 2019). Particularly, such techniques are said to have considerable advantages over unimodal ones for the analysis of music, as several music processing tasks – including similarity computation, classification in high-level categories describing emotion or expressivity, structural segmentation, and others – can benefit profoundly from multimodal approaches (Simonetta et al., 2019).

With the increasing availability of music as digital data, and the development of more sophisticated computational techniques to process, analyze, and generate such data, music researchers have adopted interdisciplinary approaches centered on the manipulation of *data corpora*. In outlining what constitutes a corpus in practical terms Tremblay et al. (2019, ibid., p. 1) point out that sound corpora are different from any collection of recorded sound, as the former are “something that musicians have settled down to explore” at various timescales, from atomic particles of sound to longer sections characterized by specific salient features. They thereby suggest that a key step for the preparation and exploration of a corpus is its *decomposition* in smaller entities such as *slices* (the product of segmentation in a single dimension, usually time), *layers* (concurring entities that form musical sound), or *objects*. This last category is more loosely defined, as it refers to a portion of corpus determined by an arbitrary set of morphological characteristics. Analysis of multimodal corpora has been employed for studying several aspects of embodied expressive performance, including interactive postural analysis of violin players (Volta and Volpe, 2019), embodied interaction between humans in virtual environments (Essid et al., 2012), and expressive movement qualities in dance (Piana et al., 2016a).

In giving an overview of multimodal techniques for music content analysis, Essid and Richard (2012) distinguish between *cross-modal processing* and *multimodal fusion*. Cross-modal processing methods aim at characterizing the *relationships* between modalities. In a case study (Gulluni et al., 2011), cross-modal processing is used for the analysis of electroacoustic music that cannot be represented using conventional notation. After interviewing musicologists with expertise in electroacoustic music analysis, the authors propose an interactive method to help them decompose an electroacoustic piece into sonic objects and correlate qualitative annotations of sonic objects with audio data. Their system aids the analysis of a given piece by: segmenting it through onset detection; asking the musicologist to assess the segmentation and label the sonic objects they want to analyze; and training a classifier to spot instances of the sonic objects on the recording. Finally, the musicologist selects and validates the results of the analysis, repeating the interaction until they are satisfied with the results. This helps with analysis tasks such as finding all the instances of a specific sound object in the piece, some of which might be difficult to hear as they might be masked by other sounds. This is an example of third-person computer-aided qualitative analysis, where human observations are correlated with audio signals by means of machine learning algorithms. In other instances, cross-modal processing might be aimed at correlating two different modalities such as the movement of performers and sound features (Caramiaux et al., 2011; Nymoen et al., 2013) or audio and video features (Gillet et al., 2007).

Multimodal fusion methods instead aim at efficiently combining the data from different modalities into a common feature representation. This process is also known as *early integration*, as features from different modalities are integrated into a multimodal feature before analysis. A common approach for feature fusion is to use dimensionality reduction algorithms – such as Principal Component Analysis (PCA; Hotelling, 1933) and Self-Organizing Maps (SOM; Kohonen, 1982), which were also employed for the design of data-driven music systems for the interaction with sound corpora (Roma et al., 2019). Moreover, research on multimodal machine learning (Baltrusaitis et al., 2019) shows that models that can relate data from multiple modalities might allow to capture complementary information that is not visible in individual modalities on their own.

This delineates a scenario where computational music analysis can harness cross-modal processing and multimodal fusion methods to shift the focus toward the *relationships* that tie together different modalities in multimodal data corpora, thereby revealing the links between low-level features and high-level expressive qualities as well as giving a new insight of structural phenomena of music performance such as chunking and coarticulation.

## Materials and Methods

This section, structured in four parts, provides an outline of the state of the art in methods for research on music performance, with the aim of considering how current qualitative and quantitative approaches can be combined in order to allow for multimodal data collection and analysis. We further define the knowledge gaps and describe the design of the pilot study.

### Qualitative Analysis

Qualitative analysis of musical performance demands a systematic approach to interpretative layers which can be described from first-, second-, or third-person perspectives. Our definition of these perspectives is closely related to those put forth by Leman (2008), but we differ substantially in our definition of the third-person perspective. For Leman, this entails only data created through quantitative measurement (see e.g., Leman, 2008, p. 80), while in the present study, qualitative data from a third-person perspective may be collected through observation, for instance, through video documentation.

#### Stimulated Recall

Stimulated recall is a common qualitative research method in education, medicine, and psychotherapy. Coined by Bloom (1953), the method was first tested in a study that used audio recordings of classroom teaching as stimuli to allow students to relive the original experience and give accounts of their original thought processes. In music research, early applications of a stimulated recall are found in studies of collaborative processes (Bastien and Hostager, 1988, 1992; Bastien and Rose, 2014). The use of stimulated recall in the present study is a further development of methods developed in music research, drawing on gesture analysis as a component in the coding process, wherein the insider perspective of a performer has been essential (see further Coorevits et al., 2016; Gorton and Östersjö, 2019; Östersjö, 2020). In their adaption of these methods for the purposes of a multimodal study of music performance, two procedures were important. First, that the video was coded by all four participating researchers, hereby aiming at creating an intersubjective understanding of the data – what Leman (2008) refers to as a second-person perspective – using open coding (see further below), and second, that descriptive analysis was added using more extensive verbal annotations. Through these steps, which were repeated several times, a structural analysis could be drawn from the coding process, while a more in-depth set of first-person observations were captured through the annotations.

The present study emphasizes how each subject involved in a stimulated recall analysis will engage in the process by activating their listening habitus (Becker, 2010, p. 130), which entails “a disposition to listen with a certain kind of focus.” We are interested in how each musician has been socialized into particular ways of listening, as well as into particular forms of performative interpretation of scored music.

#### Open Coding

Open coding is a basic procedure in grounded theory, wherein the aim is to generate “an emergent set of concepts and their properties that fit and work with relevancy to be integrated into a theory” (Glaser, 2016, p. 109). Rather than starting the analysis from a predetermined theoretical grid, the aim of open coding is to let an analytical understanding emerge from the data. Through this process, “the researcher discovers, names, defines, and develops as many ideas and concepts as possible without concern for how they will ultimately be used. How the issues and themes within the data relate must be systematically assessed, but such relationships can be discovered only once the multitude of ideas and concepts it holds have been uncovered. Turning data into concepts is the process of taking words or objects and attaching a label to them that represents an interpretation of them” (Benaquisto, 2008, p. 581). However, although it is important to approach the data “in every possible way” (Glaser, 2016, p. 108), the openness at this stage is not without boundaries. It is also necessary to bear in mind what the study itself researches, and the aim is for the coding process to gradually delimit the scope so that the codes become more structural and less descriptive.

#### Laban Movement Analysis

Laban Movement Analysis, developed from the work of Laban (1963) is widely used for describing motion qualities, particularly in dance, but also well-suited for other types of non-verbal communication. Fdili Alaoui et al. (2017, p. 4009) characterize LMA as “both a somatic and embodied practice as well as an observational and analytical system.” LMA has been successfully applied to the observational analysis of the musician’s expressive bodily movements (Broughton and Stevens, 2012). In recent years, machine learning algorithms have been employed to recognize LMA qualities in motion capture data (Silang Maranan et al., 2014; Fdili Alaoui et al., 2017; Truong and Zaharia, 2017).

### Quantitative Analysis

The premise that music is a multimodal phenomenon has led to empirical interdisciplinary studies aimed at gathering quantitative evidence of bodily engagement in musical experience. Technologies such as infrared motion capture have allowed researchers to observe human movement in detail, extracting precise kinematic features of bodily movement. This brought about a series of studies where motion analysis is based on the computation of several low-level descriptors – or movement features – linked to musical expression (Godøy and Leman, 2010). For example, acceleration and velocity profiles have been adopted for the study of musical timing (Goebl and Palmer, 2009; Glowinski et al., 2013; Burger et al., 2014; Dahl, 2015). Quantity of motion has been related to expressiveness (Thompson, 2012) and has been used to study the dynamic effects of the bass drum on a dancing audience (Van Dyck et al., 2013), while contraction/expansion of the body has been used to estimate expressivity and emotional states (Camurri et al., 2003). More advanced statistical methods, such as functional PCA and physical modeling, have led to mid-level descriptors, including topological gesture analysis (Naveda and Leman, 2010), curvature and shape (Desmet et al., 2012; Maes and Leman, 2013), and commonalities and individualities in performance (Amelynck et al., 2014).

Objective assessment of movement behavior includes measurement of kinematics (i.e., position and movements of the body and the instrument), kinetics (i.e., forces involved in the movement task), and muscle activation (e.g., onset, offset, and amplitude of muscle activity) (Winter, 2009). Various measurement systems have been used for assessments of three-dimensional motions in musical performance, including infrared high-speed optoelectronic (camera) systems (Gonzalez-Sanchez et al., 2019), inertial measurement units (IMU; Visi et al., 2017), and ultra-sonic system (Park et al., 2012b). Kinetic assessments have used force or pressure sensors for body contact with instruments, such as finger (Kinoshita and Obata, 2009) and chin forces (Obata and Kinoshita, 2012) and weight distribution (Spahn et al., 2014) in violin playing. Assessments of muscle activation commonly involve electromyography (EMG) using surface electrodes for superficial muscles (Park et al., 2012a; Gonzalez-Sanchez et al., 2019), but also fine wire electrodes to assess deeper muscle layers (Rickert et al., 2013). In musical performance, many studies have shown variation in kinematics linked to different expressive conditions (Dahl and Friberg, 2007; Weiss et al., 2018; Massie-Laberge et al., 2019).

### Knowledge Gaps

There have been attempts to link qualitative and quantitative methods in musical performance research, by integrating a performer-informed analysis (Desmet et al., 2012; Coorevits et al., 2016), an approach described by Leman (2016) as a combination of top-down and bottom-up perspectives. However, there is still a lack of coherent, systematic methods for combining computational approaches to the analysis of musical expression with qualitative analysis, informed subjective accounts, and socio-cultural perspectives (Coessens and Östersjö, 2014; Crispin and Östersjö, 2017; Gorton and Östersjö, 2019). The aim of the method development, outlined in the present paper, is to better understand how qualitative research methods, such as stimulated recall and open coding, can be further developed in order to generate data useful for the analysis of embodied musical expressivity.

The first challenge is the development of methods for multimodal data collection built on a consolidated procedure for the inclusion and integration of performer-centered perspectives on musical performance. The second challenge is to employ the resulting multimodal data corpora and take full advantage of the computational methods for multimodal analysis introduced in section Multimodal Music Representation and Analysis. This would enable new analytical approaches as well as extended, data-driven musical (and cross-disciplinary) practices (Green et al., 2018).

### Design of the Pilot Study

**Figure 1:**
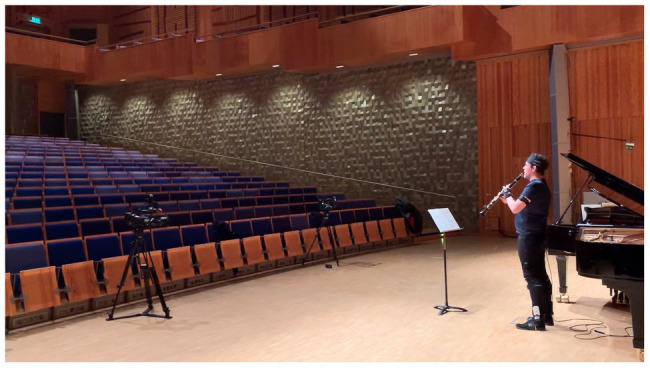
Ecological setting of the study: Studio Acusticum Concert Hall.

To develop and evaluate methods for collection and analysis of multimodal data, we chose to focus on Alban Berg’s *Vier Stücke* op.5 (Berg, 1913), performed by two members of Norrbotten NEO. The clarinet player, Robert Ek, also co-author of this article, performed the piece together with pianist Mårten Landström and was then engaged in a qualitative study carried out in a series of steps, as described below. To achieve ecological validity, the recordings took place in the Studio Acusticum Concert Hall, a recurring venue for the Norrbotten NEO ensemble (see Figure 1). Berg’s piece is a post-tonal set of miniatures. Each movement is very short but contains rapid shifts of tempo and the range of the clarinet part is 3.5 octaves which contribute to the expressiveness of the music. We also found the condensed format and the post-romantic expressiveness apt for a study of musical shaping through a multimodal analysis.

#### Quantitative Data Collection

**Figure 2:**
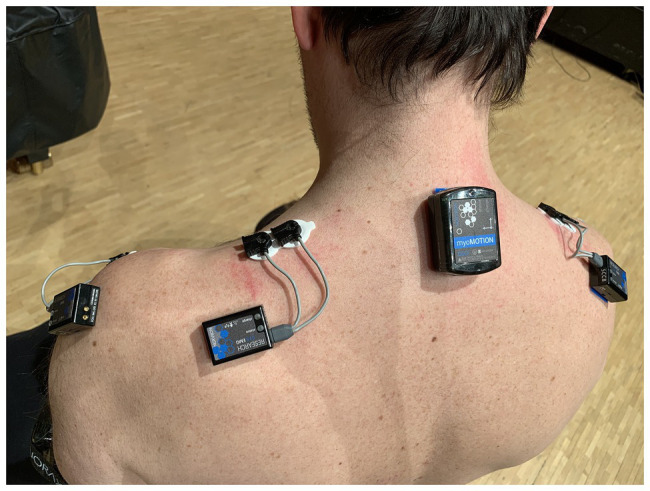
Sensor placement on the clarinetist’s back and shoulders.

Since sound-producing and sound-facilitating movements (Godøy, 2008) of clarinet performance are less visually detectable due to the affordances of the instrument, we opted to record EMG data. This allowed us to capture finger movements, and thereby study the role of sound-producing gesture in the segmentation, or chunking, of the music in the clarinet part. To quantitatively capture a comprehensive view of the movement behavior, we included measurement of kinematics, kinetics, and muscle activity using a mobile movement science lab (Noraxon, United States). We recorded audio (four channels: separate clip-on condenser microphones for clarinet and piano and a stereo recording of the hall ambience) and video of a performance (two cameras placed on the left and on the right of the stage). At the same time, we gathered data from 16 inertial sensors, six EMG electrodes, and two insole pressure sensors worn by the clarinet player (see Figure 2).

##### Kinematic Data

Full body kinematics were measured with a wireless MyoMotion (Noraxon, United States) system comprising 16 sensors based on IMU. Sensors were mounted on the head, upper arms, forearms, hands, upper thoracic (spinal process below C7), lower thoracic (spinal process above L1), sacrum, upper leg, and lower leg and feet. Sampling rate was set to 100 Hz.

##### Kinetic Data

The ground reaction force from the feet was measured bilaterally with wireless pressure sensor insoles (Medilogic, Germany), with a sampling rate of 100 Hz.

##### Muscle Activity

Muscle activity was measured with EMG using a wireless eight-sensor system, Noraxon MiniDTS (Noraxon, United States). Skin preparation was done according to SENIAM,^2^ including shaving and rubbing with chlorhexidine disinfection. Bipolar, self-adhesive Ag/AgCl dual surface electrodes with an inter-electrode distance of 20 mm (Noraxon, United States) were placed on flexor digitorium (Blackwell et al., 1999) and anterior deltoids and upper trapezius as described by SENIAM bilaterally. Sampling rate was 1,500 Hz.

#### Qualitative Data Collection

The qualitative analysis was carried out by the clarinetist, Robert Ek, in interaction with members of the research team. The analysis followed a series of steps, oscillating between first‐ and third-person perspectives (see above). An initial process of stimulated recall, using open coding had already been carried out on an earlier recording of the same piece. From this process, a series of codes that pertained to movement had emerged, through continued re-coding carried out through further intersubjective analysis by Ek, Östersjö, Visi, and the choreographer Åsa Unander-Scharin. In the stimulated recall sessions in the present study, the same descriptors were used in the descriptive analysis of movement (phase two below). The analysis was carried out in four steps, out of which the later three were designed as stimulated recall sessions using the audio and video recording as stimuli:

- To annotate the score and mark phrases, sub phrases and goal points;
- To make annotations of technical descriptions of movement;
- Analysis of movement qualities using the LMA framework; and
- Annotation of musical intentions.

##### Phrasing and Goal Points

Prior to the stimulated recall, the performer was asked to mark the score with intended phrasing and the goal points within the phrase structure. This procedure is closely aligned with what Leman (2016, p. 59), describes as the top-down perspective of a performer-inspired analysis, with the aim of providing “an understanding of the musical structure as a performer’s action plan.” What the present study adds to Leman’s approach is the performer’s further analytical engagement through stimulated recall. These data were manually transferred to ELAN (2020), and constituted an important reference point when comparing quantitative layers of data to the intended musical shaping (Coorevits et al., 2016; Östersjö, 2020).

##### Observational Analysis of Movement

The next step, carried out by Ek, was to identify and describe body movement in the performance captured in the video. Particular attention was also directed toward the coarticulation of gesture in performance, and how these structures can be understood as either spatial or temporal (Godøy, 2014). As mentioned above, the technical descriptors of movement applied in the analysis at this stage were formulated during the analysis of the previous recording of the same piece. Further observational analysis lay the ground for the next step, which involved a more systematic description of movement qualities.

##### Laban Movement Analysis

In this pilot study, we selected some aspects of the LMA framework for the purpose of categorizing expressive movement qualities. The LMA system consists of four categories – Body, Effort, Space, and Shape – and provides a rigorous model for describing and analyzing movement. The Body category describes structural and physical characteristics of the human body while moving. This category is responsible for describing which body parts are moving, which parts are connected, which parts are influenced by others, and general statements about body organization. Effort is a system for understanding the more subtle characteristics about movement with respect to inner intention. Space represents where the body is moving and the relationship between the body and the surrounding environment.

Studd and Cox (2013) describe the effort as “the dynamic or qualitative aspects of the movement. […] Effort is in constant flux and modulation, with Factors combining together in different combinations of two or three, and shifting in intensity throughout the progression of movement” (Studd and Cox, 2013, p. 159).

Effort is divided into four factors as follows:

- **Space Effort** considers focus or awareness, ranging from *direct* to *indirect*.
- **Weight Effort** considers pressure, force, or sensitivity, ranging from *strong* to *light*.
- **Time Effort** considers speed or slowing of the pace, ranging from *quick* to *sustained*.
- **Flow Effort** considers the control of movement, ranging from bound or controlled to free or released.

Effort elements usually occur in combination. While a full Effort action would consist of all four elements, it is more common to find only two or three. Each Effort factor is thought of as a continuum with two opposite ends, called elements, in which movement can vary and thus reveal different “Effort qualities.” The combination of Space, Time, and Weight is called Action Drive and comprises eight different combinations, all understood as goal-directed actions (Broughton and Stevens, 2012). Since the Effort actions are closely related to dance gestures, we decided to delimit the LMA observations to the Action Drive. In the coding sessions, Ek would carry out third-person observational analysis, employing the Action Drive categories in the coding.

##### Annotation of Musical Intentions

The use of qualitative annotations in stimulated recall from first‐ and second-person perspectives has been developed and tested in different contexts (Coorevits et al., 2016; Östersjö, 2020). While several of these earlier studies have explored intersubjective meaning formation, in the present study, Ek would mainly focus on first-person perspectives in the annotations. The qualitative analysis of video, using stimulated recall, departed from the video recordings, and the first round of stimulated recall was carried out using open coding. We outline in greater detail below how this procedure was expanded through cross-comparison of the multi-modal data collected in the study.

##### Assessment of the Data Collection Through Cross-Comparison

The first cycle of qualitative analysis was carried out by Robert Ek from the video recordings, prior to viewing any of the quantitative data. The coding and annotations were assessed by way of joint observation by the research team and further explored through cross-comparison with the quantitative data. The observations made were then the source for designing new stimulated recall sessions with Ek. These layers of qualitative coding were then synthesized, and again cross-compared with the quantitative data. Preliminary findings from the qualitative analysis, and some observations from the comparison with the quantitative data, are discussed in section First-Person Observations and Cross-Comparison of Data below.

## Results of the Pilot Study

The results of the pilot study are structured in two parts. In section Identification and Extraction of Relevant Features, we outline the methods used for feature extraction. In section First-Person Observations and Cross-Comparison of Data, we discuss the interrelation between the different types of data. We further assess the combined qualitative methods and present some examples of how the first-person annotations by the clarinetist have provided musically meaningful results, which, we will argue, have a bearing on the study of chunking and coarticulation.

### Identification and Extraction of Relevant Features

**Figure 3:**
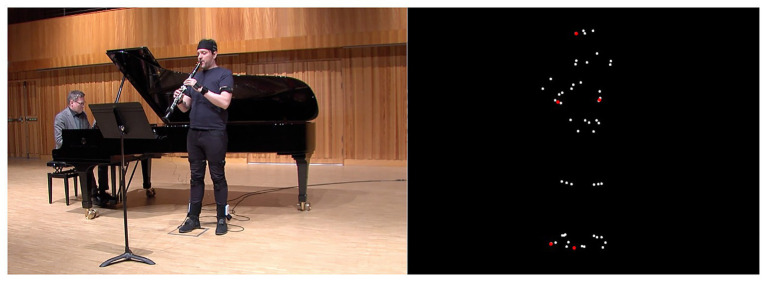
Frame of the right-side camera video feed and corresponding motion data frame showing point locations. The markers in red were used for feature extraction.

The research team worked jointly at identifying relationships between the quantitative data, structural elements in the piece, and the qualitative data obtained through the coding sessions and annotations. We computed a set of features from the recorded quantitative data in order to cross-compare it with the qualitative annotations and identify patterns, correlations, discrepancies, etc. From the motion data, measured with the IMU system, we selected five of the 53 trajectories obtained by processing the inertial data: the body center of mass, the left and right elbows, the left and right toes, and one trajectory for the head, highlighted in red in Figure 3. We then computed the magnitude of a jerk for each of these trajectories. Jerk is the rate of change of acceleration, and it has been linked to musicians’ expressive intentions (Dahl and Friberg, 2003). Peak detection was used to spot local maxima in the jerk values.

Another feature we extracted from the motion data is the Contraction Index (CI). CI is calculated by summing the Euclidean distances of each point in a group from the group’s centroid (Fenza et al., 2005). When used with full-body motion capture, it is an indicator of the overall contraction or expansion of the body, and it has been used for emotion recognition applications (Piana et al., 2016b). We computed CI for each frame by summing the Euclidean distances between all the points and the center of mass of the body. We then used peak and trough detection to mark CI local minima and maxima, which respectively correspond to moments in which the body is relatively contracted and expanded.

The data obtained from the insoles gave us an estimate of how the weight was distributed on Ek’s feet at any time during the performance. To better understand the dynamics of weight shifting – which has been used for the analysis of expressive movement qualities (Fdili Alaoui et al., 2015) – we calculated the difference between the weight on the left foot and that on the right foot. This measure is therefore equal to zero when body weight is equally distributed between left and right foot, positive when there is a relatively higher load on the left foot, and negative when there is a relatively higher load on the right foot. The derivative of this measure therefore indicates how quickly Ek shifted his body weight during the performance. Additionally, we summed up the left and right weight values to obtain an estimate of the overall vertical acceleration dynamics. This measure showed when the performer pushed himself upward against gravity (e.g., if the performer were to perform a jump, the data would ostensibly show a peak during the initial thrust, then a trough as the body takes off, and then a second peak on landing). In the data, we observed correspondences between sharp troughs in this measure with annotations of gravity and energy, as well as with Direct/Quick/Light (DQL) LMA movement qualities.

We computed the root mean square (RMS) of the EMG data of the anterior deltoids and the finger flexors after bandpass filtering (low frequency = 20 Hz; high frequency = 350 Hz) to reduce signal noise. The resulting values are an estimation of muscular activation of the finger flexors and anterior deltoids during the performance. The data were further processed to find abrupt changes and to spot onsets and offsets of muscular activation. We observed correspondences between the onsets and offset of the finger flexors and indicators of phrasing in the annotations, while the activation of the anterior deltoids corresponded with increases in the CI values, as the activation of these muscles is linked with rising the elbows.

In order to obtain a measure of loudness of the clarinet sound, we computed the RMS values also of the audio, recorded from the clip-on microphone placed on the clarinet. The peaks in the resulting loudness envelope often corresponded to troughs in the weight sum measure obtained from the insoles as explained above, particularly while approaching annotated goal points, indicating that the integration of these features might be useful for segmentation and individuation of goal points.

### First-Person Observations and Cross-Comparison of Data

**Figure 4:**
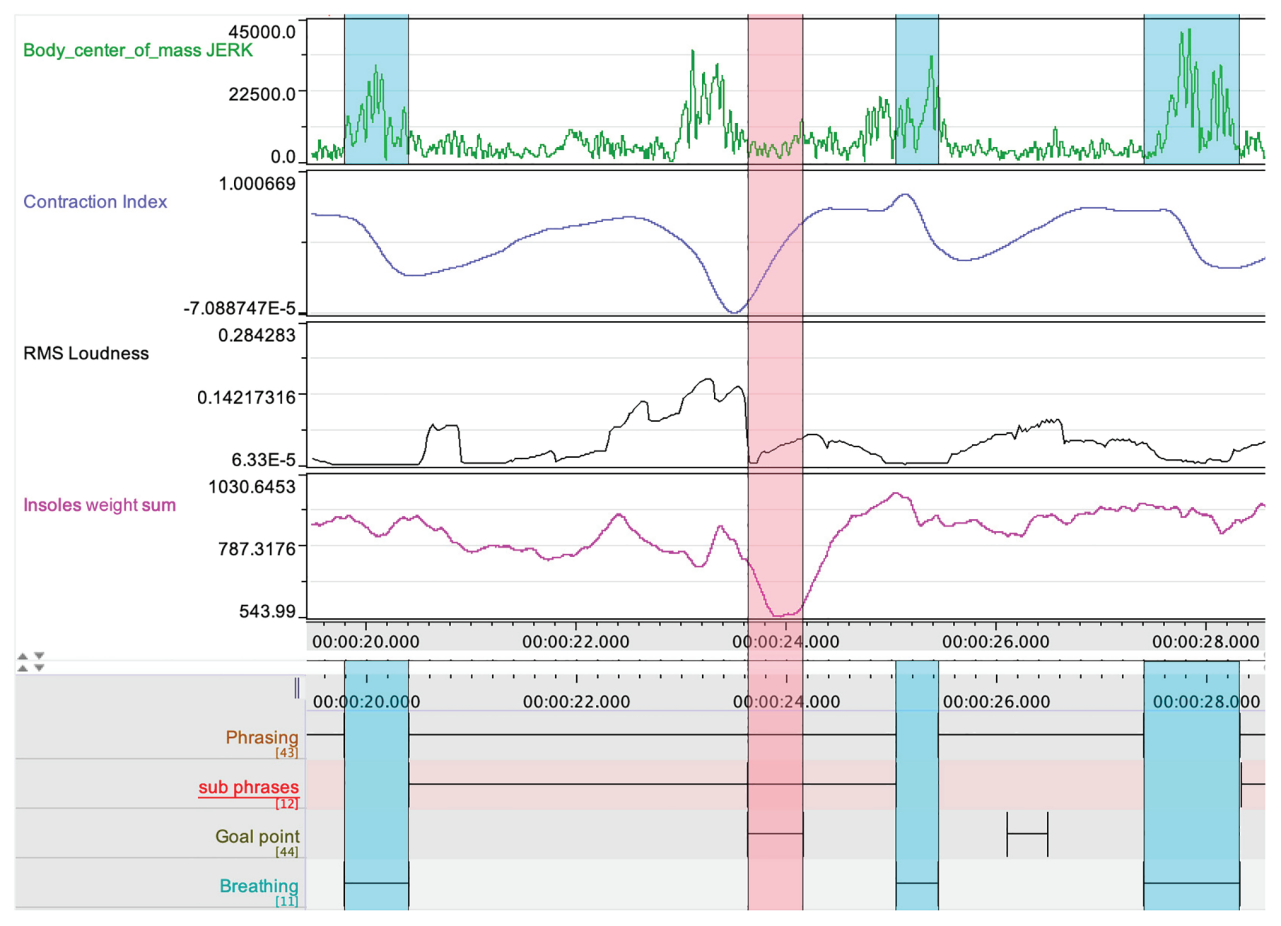
A segment of the multimodal recording showing jerk, CI, loudness, and insoles weight sum, which displays the coarticulation of body parts in relation to a goal point, indicated by the red rectangle. The blue rectangles indicate the breathing, such as captured also in the jerk data.

For the purposes of this pilot study, it was essential for the research team to observe and explore possible confluences between the different data streams. In particular, we wished to assess the relation between certain patterns in the quantitative data and the qualitative annotations made by Ek. An example of such cross-comparison can be seen in Figure 4. Here, we can see a striking mirroring pattern between the loudness of the clarinet sound and the curve of the insoles weight sum – suggesting a relation between the vertical thrust in the performer’s body movement and the dynamics in the musical performance. Further, we also see how the CI, jerk, and insoles weight sum coincide in the prefix to the goal point indicated in the initial stage of the qualitative analysis.

The final layer of qualitative analysis was again carried out by Ek in the form of a stimulated recall. Here, the research team’s cross-comparison of different constellations of quantitative and qualitative data from the study, relating them to the musical content, was central. This cross-comparison was carried out to explore the possibility of enhancing the qualitative findings through the use of stimulated recall sessions using the video data, by also asking Ek to reflect on commonalities and discrepancies between his annotations and the quantitative data. In the following paragraphs, we provide four examples of how further detailed understanding could be drawn out of these multimodal sources.

**Figure 5:**
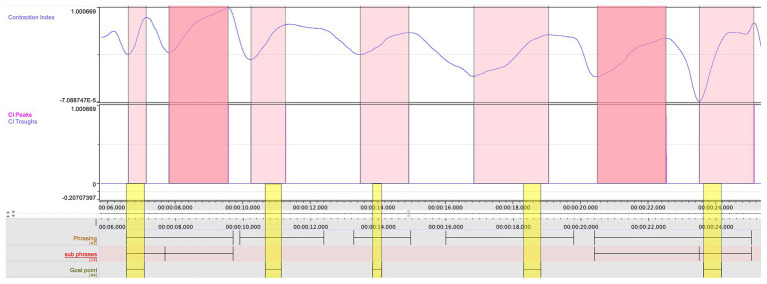
The CI, aligned with the annotated phrasing, and goal points (marked in yellow) in the first 20 s of the first movement. Each rising curve in the CI is marked in red, and the two instances in which the CI does not lead to a goal point are darker.

First, when looking at the CI in the first movement, computed from the quantitative analysis (see Figure 5), and comparing it with the annotations from the qualitative coding, certain connections were observed by the research team. The troughs followed the overall gestural shape in the music of the first movement and, upon closer examination, it reveals that almost all annotated goal-points occurred when the CI was rising (i.e., indicating that the movement span is expanding in relation to the center of mass). A few deviations from this pattern attracted the attention of the research team, and Ek was invited to make a closer examination of these instances, through a new round of stimulated recall. His observations were documented in new qualitative annotations. This renewed qualitative analysis was fruitful in evoking musically meaningful observations. The first instance concerned the opening phrase in which Ek had annotated a goal-point right at the beginning. But here, there are two rising curves in the CI, and the second one does not lead to an annotated goal point, Ek had annotated a goal point located right at the beginning of the phrase. When again exposed to the video recording, Ek entered the following annotation:

I suddenly realize that this phrase always [has] been awkward for me to play, it always feels disembodied. My professor at the university wanted me to grab the music from the air interpreting it as being the middle of the phrase and then finish the phrase. The embodied gesture coupled with the quantitative data reveals that I make a poor job and my feeling of disembodiment turned out to be true. With this in mind, I will reinterpret the first phrase next time I play this piece.

**Figure 6:**
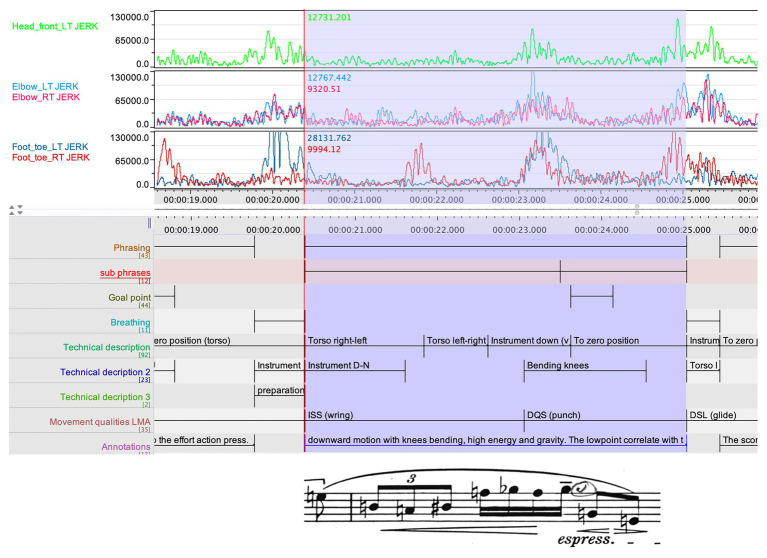
Three layers of the jerk values from several body parts, aligned with the phrasing data, and with the clarinet part of the relevant phrase at the bottom.

Hence, Ek divided the phrase in two sub-phrases in which the second sub-phrase holds the part with the second rising curve in the CI. Although there was no annotated goal point, in accordance with the above annotation, Ek now realized that his interpretation entailed a second goal point in this phrase, although his teacher’s instruction had made it hard for him to identify this. The second instance where the CI does not align with a goal point is around 20 s (see Figure 5). Here, we find an increase in the CI but, for the second time, the increase in the index does not lead to an annotated goal point. In Ek’s annotations in the score, the phrase is divided in two sub-phrases, and the increase in the CI marks the end of the first sub-phrase. The research team was, however, still uncertain of what the rising CI represented in the performer’s shaping of the phrase. We had already been cross-comparing the jerk values with the phrasing, and here, this data appeared to hold a clue. In Figure 6, we see a summary of the jerk values from several body parts, aligned with the phrasing data, and with the clarinet part of the relevant phrase added in.

The data clearly indicates a temporal coarticulation in which the different body parts initially are not aligned, but all come together on the third beat, which Ek had marked as a goal point in the score. Hence, the second rising CI which did not align with an annotated goal point (see Figure 5), marks the initial impetus in a longer trajectory in the musical shaping. When this observation had been made by the research team, Ek again viewed the video and made the following annotation:

Structurally, this goal point is of a higher order than the previous ones, and is the first culmination of the material introduced in the first bar. This is also indicated in the score, since this is the first instance of a joint chord on downbeat in the two instruments. But what concerns me in the shaping of this phrase is to achieve an elastic shaping of the phrase, up to this goal point. The jerk data made me see how my intentions for phrasing are in fact represented in the complex relation between body parts, moving, as it were, with different trajectories toward the common goal point.

Ek’s observations of perceived movement qualities, using the LMA framework, also coincide with the activity in the jerk values (see Figure 6). In the first part of the phrase, the movement is categorized as Indirect/Sustained/Strong (ISS), while in the preparation for the goal point, the movement is annotated as Direct/Quick/Strong (DQS). This set of observations of chunking and coarticulation constitutes our second example.

**Figure 7:**
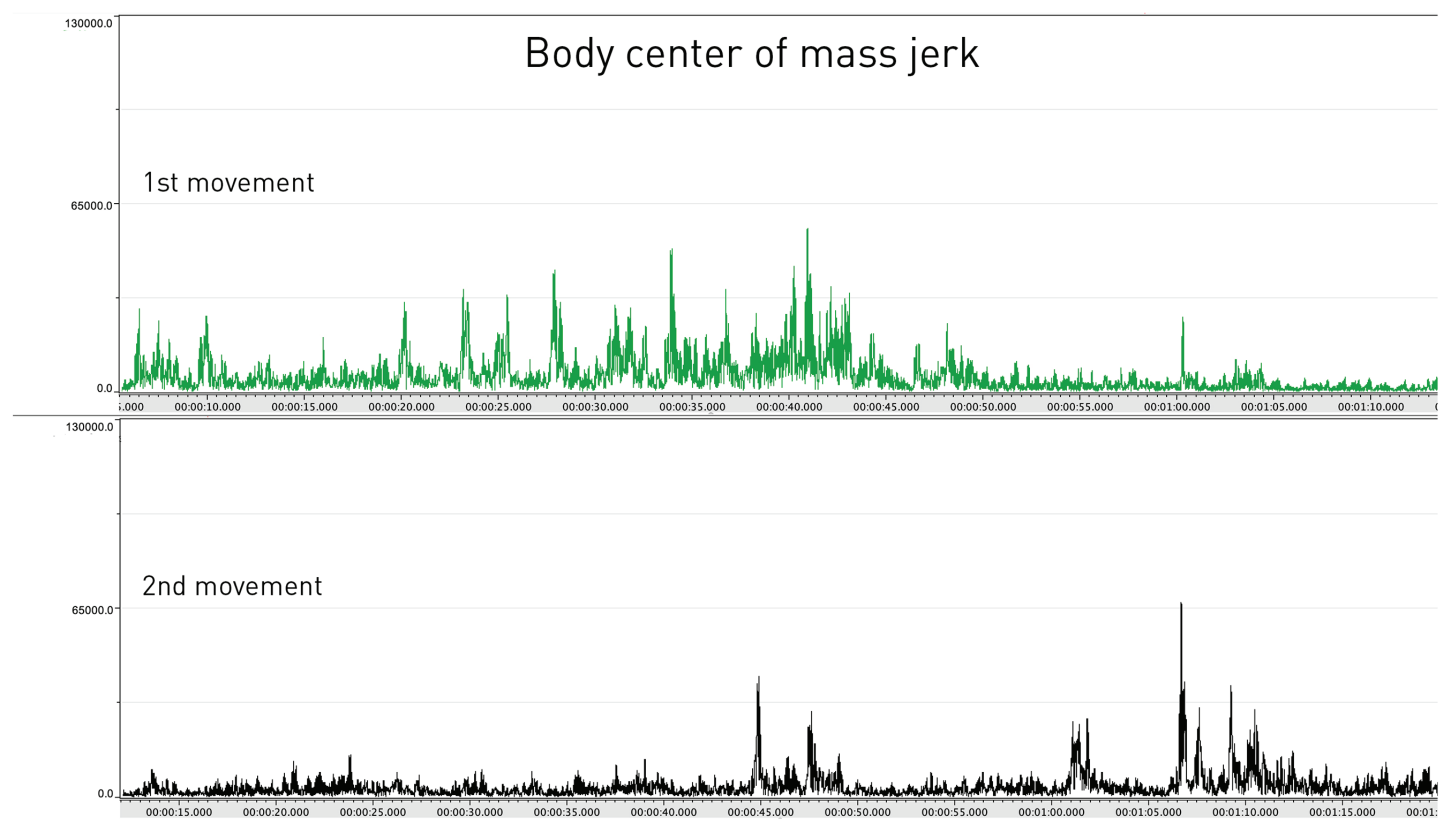
The body centers of mass jerk values in the first and second movements.

**Figure 8:**
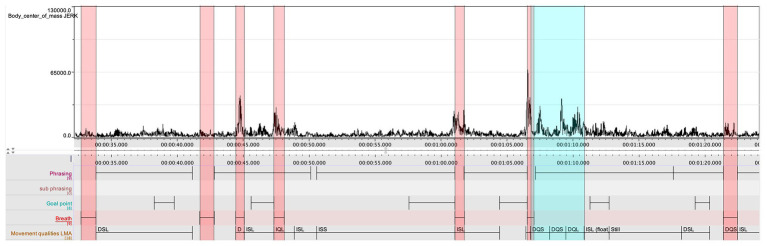
A representation of the jerk values in the second movement, with the breathing marked with red rectangles, and two peaks in the jerk data marked with blue rectangle.

In the comparative analysis, the research team aligned the jerk values of the clarinetist’s center of mass from movements 1 to 2 (see Figure 7). A comparison between the two movements showed that the second movement had lower jerk values on average. This was expected, as the second movement is slower and with a more limited dynamic range compared to the first. However, it was also striking that the second movement had the highest peaks in the jerk data. After marking the occurrence of each peak in the score in both movements, we noticed that nearly all the peaks corresponded with breathing, which is typically carried out at the prefix to a new phrase (see Figure 8). If we return to Figure 4, a further observation can be made. Here, in the three instances when they coincide with breathing (marked with blue rectangles), we see how the peaks in the jerk data coincide with low amplitude in the RMS loudness. The second peak in the jerk data in which the RMS loudness is instead high, does not represent breathing, but rather the performer’s preparation aimed at the goal point. This interplay between different modalities can be systematically harnessed by means of machine analysis, further expanding the potential for a holistic understanding of music performance.

**Figure 9:**
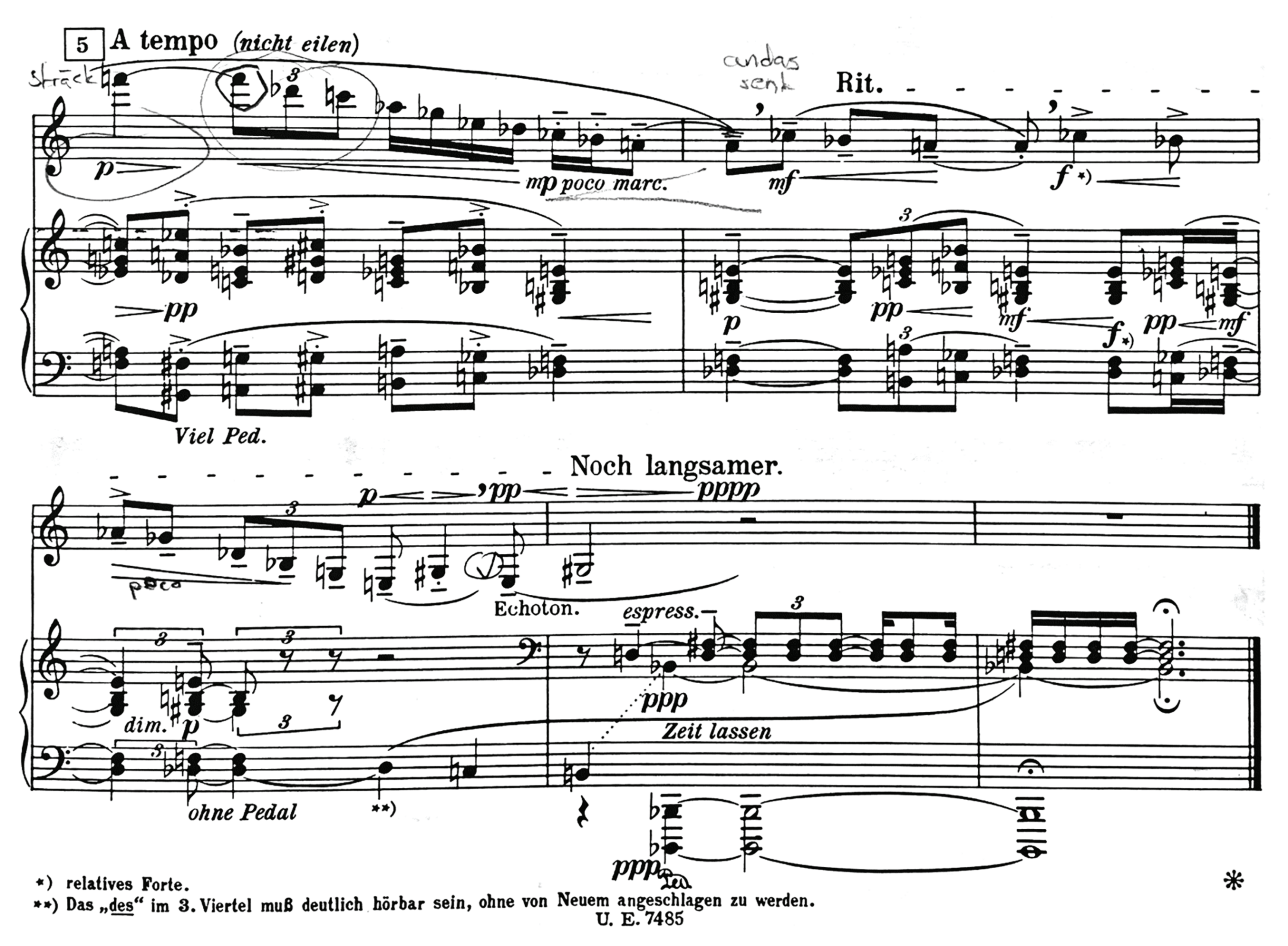
Bars 5–9 of the second movement in Alban Berg’s *Vier Stücke* op.5 (Berg, 1913, p. 5)

The highest peaks in the jerk values in the second movement, found in bar 6 (see Figure 9), seemed to demand further study, and Ek was asked to return to the second movement for a new session of stimulated recall. When reviewing the video recording, he realized that the highest peak did not merely represent a quick and deep breath, which is motivated by the length of the following phrase, but furthermore, reflects the musical phrasing.

In the score, the clarinet starts out with a three-note figure in eight notes, and, after the third beat, the first notes, a Cb and a Bb are repeated, now in *forte*, accentuated and with a crescendo leading up to the next downbeat. The downbeat in bar 7 was annotated by Ek as a goal point, which seems to be a logical aim, given the notated structure.

However, when Ek revisited the data, and the video recording, he made the following set of observations:

It is clear from the extensive prefix to the second iteration of the Cb, captured in the jerk values, that I aim at the Cb in this bar. It also is by far the loudest note in the phrase. This may have multiple reasons, since the Bb and Ab is so much weaker on the clarinet than the Cb. They are in the so-called throat register, and hence, I shift register between the Cb and the Bb. Also, the piano has a crescendo which starts on the second and leads up to the fourth beat, which provides a clear direction for the entry of the second Cb in the clarinet. While the structural downbeat on the beginning of the next bar certainly guides our phrasing, perhaps partly due to the weakness in the register of my instrument, I compensate for the lack of dynamic force by speeding up toward the Ab. At the same time, this also gives a natural shape to the closure of the phrase. Still, it was only when studying the jerk data that I realized that in my rendering of this phrase, again, perhaps due to the limitations of the instrument in this register, the greatest intensity was not by the intended goal point, but in the lead to it.

The LMA coding by Ek is very much aligned with the jerk data discussed above (see Figure 8), and casts further light on the shaping of the entire phrase. The two first peaks in the jerk data, starting in bar 6 (marked with blue in Figure 8) occur straight after the breath. They were annotated with DQS, and the third was annotated with DQL. Hence, the downbeat, which should have constituted the highpoint, was annotated as “light,” while the two preceding as “strong.” When the energy begins to dissolve, the LMA annotation is Indirect/Sustained/Light (ISL), which in turn leads from an annotated “zero position” to “still.” Hence, when annotating the movement qualities, Ek made observations that confirmed the insight he later obtained when doing the final stimulated recall. If the agency of the instrument is understood as a contributing factor in his rendering of the phrase, then it should also be noted that the negotiation between performer and instrument can be observed also in the movement qualities, and in particular in the shift from “Strong” to “Light” in the LMA-annotations. A similar representation of performer-instrument interaction in the shaping of the music is found in the final bars of the first movement. The music culminates in bar 8, and the clarinet then gives shape to a final melodic figure, which starts on the second beat of bar 9. The final note, an A, is then repeated across the two final bars (annotated in the score to be performed “ohne ausdruck,” with a notated ritardando starting in bar 10).

**Figure 10:**
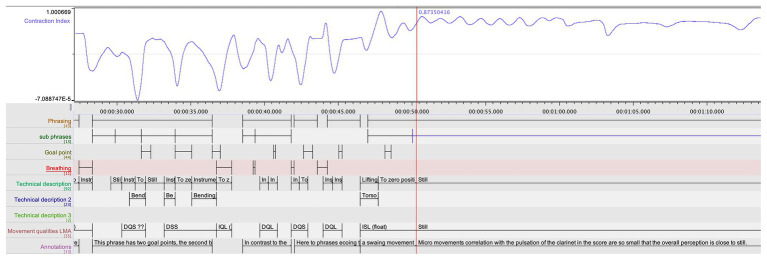
The CI in bars 6–12 of the first movement.

Some patterns in the CI of the entire section (bars 6–12) in the first movement can be connected to the musical shaping of these bars (see Figure 10). Each time the CI makes a quick dip, we encounter an annotated goal point. Just as in the previous example, the bodily action is closely aligned with the prefix to the goal points, with the CI typically connected with the clarinetist bending his knees. This pattern is ongoing through the continuous build-up, all the way up to bar 8, after which the low points in the CI gradually decrease, throughout a longer diminuendo. This process is in turn followed by a coda in which the clarinet gradually moves to a repeated A, first articulated as pulsating eighth notes, and then slowing down and bringing the movement to a close. Here, the CI marks a clear shift, and also provides an image of the pulsations (largely marked by movements of the elbows) and the structural ritardando. But what attracted the attention of Ek, when he studied the index, is how he found that the overall CI was higher than what had been recorded as his “neutral” position. When he reviewed the video he made the following annotation:

This section is marked “*ohne ausdruck*” and I had sought to create such an expression. However, when considering the elevated and widened bodily position, suggested by the CI, and reviewing the video (at the point where I lift the bell and keep my head high), I realize that my posture is not “neutral.” In retrospect, I find that my position itself projects a particular lightness to the final bars, which perhaps exceeds the indicated non-expressiveness.

Ek further noted how the perceived lightness was similar to the descriptor of “light” in the effort factor weight in LMA. But the shift in the performer’s position in these final bars is again related to the affordances of the instrument since the angle of the instrument must be consistent, across any series of movements, when the instrument is lifted, like in these final bars, the entire body must follow. A comparison between the CI of Ek’s position before the beginning of the piece (the reference “zero” position) and the final bars confirm the visual observation of the curve. The CI in the zero position is approximately 0.665 and, in the ending, 0.856. If in this final example, expressive gesture in the performance adds further quality to the interpretation, rather than merely highlighting or accompanying the musical shaping, it must also be noted that the role of the performer’s movement is shifting across the four examples drawn from this pilot study. In the first example, we see how the movement data, and the qualitative coding of musical structure, unveils conflicting ideas regarding the interpretation of the score. The second example illustrates how the coarticulation of movement, here captured in the jerk data, may align in the preparation for the goal point of a phrase. The third example is also concerned with coarticulation and indicates how breathing can be woven into the expressive enforcement of musical intentions.

## Discussion and Future Work

While the scope of the pilot study we discuss is limited to data from one single performance, some observations can be made regarding the method development it seeks to explore. We see indications that meaningful data can be drawn from stimulated recall interviews with musicians, and further, that a cross-comparison with quantitative data, recorded in the same performance, may enhance this procedure. More specifically, the results of this pilot project suggest that new perspectives on the role of coarticulation in musical performance – and also the role of embodiment in musical shaping – can be achieved through such combinations of methods. For instance, we find that added value is to be found in reflections on the agency of the instrument (as in the rendering of the lead up to the indicated goal point, discussed in example two) and through the socio-cultural perspective suggested in example one, when the role of a former teacher turns out to be directly influencing the rendition of the opening phrase in the first movement.

Clearly, the interaction between the authors in the research team was beneficial for the repeated stimulated recall sessions, but the actual qualitative analysis was mainly carried out from a first-person perspective by Ek. We now see that the oscillation between first‐ and second-person perspectives (see for instance Coorevits et al., 2016; Gorton and Östersjö, 2019; Östersjö, 2020) have benefits which we will implement in the continuation of the project.

We also wish to connect the observations made by Ek of the movement qualities in the sections discussed in section First-Person Observations and Cross-Comparison of Data, through the analytical grid of LMA, to the basic types of gestural sonorous objects (Godøy, 2006), presented in section Coarticulation, Chunking, and Segmentation in Music Performance above. There are obvious connections between the two, most immediately in the Time Effect Factor of LMA, which corresponds closely with the impulsive and sustained gestural sonorous objects. While LMA is a comprehensive system based on bodily action, the gestural sonorous object draws its typology from the study of sound objects, arguing that the multimodal nature of our perception suggests that a musician’s movements in performance should be inherently connected to the resulting sound object. It is indeed also this very connection which we seek to explore, and therefore, an analytical framework should make these connections as explicit as possible. We believe that a comparative study of these two systems might lay the grounds for an analytical framework which is grounded in a multimodal understanding of musical perception. Such a comparative study might, in itself, provide important knowledge for the development of observational analysis of musician’s movement in performance. Further, this would constitute the beginning of a development of a multimodal ontology for music analysis, expanding on the concepts developed for an ontology of audio features proposed by Allik et al. (2016), in the context of MIR. Following Avanzini and Ludovico (2019, p. 3), we believe that “the availability of music information structured in this way may allow to extract higher-level meaning using appropriate features and machine learning approaches.” In fact, this will extend the machine learning of musical gestures (Visi and Tanaka, 2020a) and enable cross-modal mapping approaches based on higher-level musical knowledge (Visi et al., 2017) as well as AI-assisted techniques for the exploration of high-dimensional data (Visi and Tanaka, 2020b).

As outlined in section Knowledge Gaps, we see two main challenges in the development of methods to systematically link quantitative and qualitative data for the multimodal analysis of music performance. The first one, consolidating a method for data collection to build a multimodal data corpus, has been approached with the pilot study presented here. At the same time, we see several avenues for further development, additions, and modifications. Future studies will address the second challenge, that is, to perform computational analysis of the resulting data corpus. As denoted in section Coarticulation, Chunking, and Segmentation in Music Performance, machine learning, and multimodal fusion constitute promising techniques for aiding the identification and mapping of phenomena such as chunking and coarticulation, particularly in a scenario where training data is augmented by qualitative annotations. Decomposition in chunks and the dynamics of coarticulation are still open problems in music research, as only a few empirical studies look at how these processes unfold, and – to our knowledge – none of these address longer time spans, or look at patterns across multiple performances. Prior studies employed computational techniques for the automated identification of movement qualities (Fdili Alaoui et al., 2017). However, this approach has not been implemented in musical performance studies, with data on chunking and analysis of gestural sonic objects (Godøy, 2018). We expect automated decomposition and segmentation techniques to benefit from the qualitative data in the corpus, but we also see how the collection and assessment of new qualitative data may take advantage of interactive tools in a paradigm similar to the work by Gulluni et al. (2009) described in section Coarticulation, Chunking, and Segmentation in Music Performance. This might ultimately lead to a two-way process in which, on the one hand, qualitative observations inform the structural relationships between qualitative data streams and, on the other, this information supports the gathering and refinement of new qualitative data.

Even though the present study is focused on the development of a method for the production and collection of qualitative data paired with multimodal quantitative data, it also highlighted the challenges related to the use of EMG signals in expressive gesture analysis. Extracting RMS amplitude, offsets, and onsets of EMG showed some correspondences with musical structures and qualitative annotations. However, given the complexity of the signal and its susceptibility to noise, we believe that further processing, the extraction of additional descriptors, and the adoption of machine learning techniques (Zbyszynski et al., 2020, forthcoming), are necessary steps to fully integrate EMG in the corpus analysis.

We have observed in several instances how important information can be drawn from quantitative measures of movement behavior, i.e., kinematics, kinetics, and muscle activity. As outlined in the result section First-Person Observations and Cross-Comparison of Data, we found both associations and diversities between features. For example, associations between CI, jerk, and forces from the insoles (insoles weight sum) as they coincide in the prefix to the goal point, and between EMG RMS amplitude of the anterior deltoids which correspond with increases in the CI values. We discuss above how peaks in the jerk data coincided with low amplitude in the RMS loudness, and how this is an indicator of breathing. We have also observed correspondences between the onsets and offset of the finger flexors EMG and indicators of phrasing in the annotations. These findings support the notion that a more comprehensive analysis can be achieved through cross-modal processing and multimodal fusion methods on quantitative and qualitative data (Essid and Richard, 2012; Lesaffre and Leman, 2020). Further work on larger datasets is necessary, and we are therefore planning further data collection involving diverse instrumentalists and instruments.

### Implications on Musician’s Wellbeing

The focus of the present study was to gather multi-layered data related to embodied musical expression, which thereby guided the choice of features calculated from the measurements of the IMU, EMG, and insole systems. Other relevant features that are commonly calculated from such measures include, e.g., kinematic measures of joint angles, and velocity and acceleration of the joints and body parts; kinetic measures of forces acting on different body parts or applying inverse dynamic analyses to kinematic measures; and muscle activity normalized to maximum voluntary contraction and muscle co-contractions. Such conventional features added to the data corpus may increase understanding of the embodied musical expression, while also having substantial use for ergonomic analyses and assessment of injury risk in future research.

We expect that the multimodal approach discussed in this paper will contribute substantially to the study of movement behavior related to the wellbeing among musicians. It has a bearing both on professional as well as educational contexts.

It is well-known that the prevalence of musculoskeletal pain conditions is relatively high among professional musicians, and especially located to the neck, back, and upper extremities (Paarup et al., 2011). Risk factors include, e.g., biomechanical factors such as repetitive movements, load-bearing, and awkward postures (Kaufman-Cohen and Ratzon, 2011). These factors can be explicitly measured and analyzed with methods outlined in the present study, and further developed through additional methods for qualitative inquiry. Increased knowledge and developments in this area can thereby contribute to better assessment methods, and as a continuation, more efficient prevention and intervention strategies to counteract health conditions among musicians.

Skilled performance has been observed to involve specific attributes regarding movement behavior, e.g., consistency, minimal effort, and flexibility (Higgins, 1991). A musician’s transition from novice to expert will typically pass various learning phases through which their performance can be seen to develop. The projected multimodal corpus is expected to help identify specific attributes or features that are characteristics of highly skilled musical performance, as well as specific features related to the different phases of learning. We expect this knowledge to be valuable in learning and teaching situations, in order to promote skilled movement behavior while minimizing the risk of injury.

### Method Refinement and Concluding Reflections

For the continued data collection, it will be necessary to develop a set of descriptors for the coding of movement that can be common for different instrumentalists, and also shared across different instrument types. Greater efficiency will be needed in every step, in order for the stimulated recall procedure to be feasible with a greater number of performers, who also will not always be participating as researchers. In order to further develop this framework, a series of similar studies with one and two performers will be carried out in the autumn of 2020. As the corpus development continues, we see the development of methods that also assess the inter-annotator agreement (Bobicev and Sokolova, 2017) as essential. Such an approach would be emblematic for a trajectory within the project, from the current focus on high-level features, toward an increasingly multimodal analysis, aiming to become as holistic as is music in performance.

## Data Availability Statement

The data that support the findings of this study are available from the corresponding author, FGV, upon reasonable request.

## Ethics Statement

Written informed consent was obtained from the individual(s) for the publication of any potentially identifiable images or data included in this article.

## Author Contributions

All authors listed have made a substantial, direct and intellectual contribution to the work, and approved it for publication.

### Conflict of Interest

The authors declare that the research was conducted in the absence of any commercial or financial relationships that could be construed as a potential conflict of interest.

## Footnotes

^1^https://norrbottensmusiken.se

^2^http://www.seniam.org/
